# The moderating role of lifestyle, age, and years working in shifts in the relationship between shift work and being overweight

**DOI:** 10.1007/s00420-020-01519-4

**Published:** 2020-02-10

**Authors:** Gerben Hulsegge, Willem van Mechelen, Heleen Paagman, Karin I. Proper, Johannes R. Anema

**Affiliations:** 1 Amsterdam UMC, Vrije Universiteit Amsterdam, Department of Public and Occupational Health, Amsterdam Public Health Research Institute, Van der Boechorststraat 7, 1081 BT Amsterdam, The Netherlands; 2 The Netherlands Organization for Applied Scientific Research, TNO, Schipholweg 77-89, 2316 ZL Leiden, The Netherlands; 3 Human Movement and Nutrition Sciences, Faculty of Health and Behavioural Sciences, University of Queensland, Brisbane, Australia; 4 Division of Exercise Science and Sports Medicine (ESSM), Department of Human Biology, Faculty of Health Sciences, University of Cape Town, Cape Town, South Africa; 5 School of Public Health, Physiotherapy and Population Sciences, University College Dublin, Dublin, Ireland; 6 Department Research and Business Development, HumanTotalCare, Zwarte Woud 10, 3524 SJ Utrecht, the Netherlands; 7 Centre for Nutrition, Prevention and Health Services, National Institute for Public Health and the Environment, Antonie van Leeuwenhoeklaan 9, 3721 MA Bilthoven, The Netherlands

**Keywords:** Moderators, Interaction, Obesity, Night work, Rotating shift system, Lifestyle behaviors

## Abstract

**Purpose:**

This study aimed to investigate the relationship between the moderating role of lifestyle, age, and years working in shifts and, shift work and being overweight.

**Methods:**

Cross-sectional data were used of 2569 shift and 4848 non-shift production workers who participated between 2013 and 2018 in an occupational health check. Overweight (BMI ≥ 25 kg/m^2^) was calculated using measured weight and height; lifestyle was assessed by questionnaires. Multiple-adjusted logistic regression with interaction terms between shift work and potential moderators assessed multiplicative interaction; the relative excess risk due to interaction assessed additive interaction (synergism).

**Results:**

Shift work was significantly related to being overweight (OR 1.53, 95% CI 1.33 1.76). The strength of this association did not differ by level of sleep quality, fruit and vegetable intake, and physical activity (*p* ≥ 0.05). Additive and multiplicative interaction by smoking status was present (*p* < 0.01), with a stronger relationship between shift work and being overweight among non-smokers compared to smokers. Older age as well as more years of exposure to shift work were, independently from each other, related to a stronger relationship between shift work and being overweight (multiplicative interaction *p* < 0.05).

**Conclusion:**

Shift work was to a similar extent related to being overweight among those with a healthy and unhealthy lifestyle. This does, however, not imply that shift workers can behave unhealthy without any harm. Based on the evident health benefits of a healthy lifestyle, it is still recommended to get sufficient quality of sleep and to meet the recommended level of daily physical activity and, fruit and vegetable intake.

**Electronic supplementary material:**

The online version of this article (10.1007/s00420-020-01519-4) contains supplementary material, which is available to authorized users.

## Introduction

Shift work that includes night work has increased over the last decades to accommodate the demands of our 24/7 economy. Currently, about 15–20% of the workforce in Europe and the US is working in shifts that also include night work (Parent‐Thirion et al.^ 2016^; Bureau of Labor Statistics 2018). Although beneficial for the economy, shift work places a large burden on the shift work population as it increases the risk of chronic conditions, such as overweight (Proper et al. 2016; Sun et al. 2018; van Drongelen et al. 2011). To alleviate the adverse health effect of shift work, it is necessary to have insight into potential subgroups of shift workers who are at greatest risk of health problems, which can be done by identifying moderators.

Lifestyle behaviors might be one of those moderators. Systematic reviews showed shift workers to have poorer sleep quality and poorer dietary patterns compared to non-shift workers (Amani and Gill 2013; Linton et al. 2015; Souza et al. 2019), and several observational studies indicated shift workers to be less physically active and to smoke more often (Loprinzi 2015; Nabe-Nielsen et al. 2011; Trinkoff and Storr 1998; van Amelsvoort et al. 2006; Vandelanotte et al. 2015). However, the way these lifestyle factors contribute to shift work-related overweight is yet unknown. As lifestyle factors are related to shift work as well as to overweight (Amani and Gill 2013; Linton et al. 2015; Loprinzi 2015; Nabe-Nielsen et al. 2011; Souza et al. 2019; Trinkoff and Storr 1998; van Amelsvoort et al. 2006; Vandelanotte et al. 2015), they may potentially interact with shift work in the relationship between shift work and overweight. Various reasons for this potentially moderating role of lifestyle exist. For example, a healthy diet and sufficient levels of physical activity with its corresponding increase in physical fitness improve individual tolerance to shift work (Atkinson and Davenne 2007; Atkinson et al. 2008; Costa 2003), which is defined as the ability to adapt to shift work without adverse consequences (Andlauer et al. 1979). A healthy lifestyle might, via a higher tolerance to shift work, protect against the negative effects of shift work on body weight (Atkinson and Davenne 2007; Atkinson et al. 2008; Costa 2003), and may, therefore, serve as a moderator in the relationship between shift work and overweight. In addition, unhealthy lifestyle behaviors and poor timing of exercise, food intake, and sleep can alter the activity of circadian clocks in peripheral tissues and desynchronize circadian rhythms (Gabriel and Zierath 2019; Lewis et al. 2018; Wehrens et al. 2017; Youngstedt et al. 2019). As a consequence, unhealthy lifestyle behaviors may increase the circadian misalignment that is already caused by shift work, which may lead to synergistic effects of shift work and unhealthy lifestyle behaviors in the development of overweight. Exploration of these potentially moderating roles of lifestyle may identify subgroups of shift workers with an increased risk of overweight.

Age and the number of years working in shifts may also moderate the relationship between shift work and overweight. Three of the four available studies in a recent systematic review found that a higher number of years of shift work was related to a higher risk of being overweight (Sun et al. 2018). As all four studies described in this review were conducted among nurses, less is known about the potentially moderating role of years of exposure to shift work in other occupational sectors with other shift schedules. The moderating role of age is also yet unclear because one review concluded that tolerance to shift work decreases with higher age (Saksvik et al. 2011), whereas another review concluded that there is no evidence for less shift work tolerance in older workers (Blok and de Looze 2011). In addition, longer exposure to shift work highly correlates with older age. These factors may, therefore, interact with each other in the relationship between shift work and overweight, as indicated by one study among offshore workers (Parkes 2002b). This study showed that years working in a relatively uncommon shift schedule rotating between seven 12-h day shifts, seven 12-h night shifts followed by 14 days off work was a more important moderator than age in the relationship between shift work and self-reported body mass index (Parkes 2002b). More research in workers with other shift schedules and objectively measured overweight is needed to elucidate whether age, years of exposure to shift work, or both moderate the relationship between shift work and overweight.

Therefore, the first aim of the present study was to investigate the moderating role of lifestyle in the relationship between shift work and being overweight. The second aim was to investigate the moderating role of age, years of exposure to shift work and their interaction in the relationship between shift work and being overweight. We hypothesized that the relationship between shift work and being overweight is moderated by lifestyle behaviors, age, and years of exposure to shift work.

## Methods

### Population

A nationwide Dutch occupational health care service continuously gathered data about work and health using standardized questionnaires and physical examinations, as part of their standard voluntarily periodical occupational health checks among workers. Cross-sectional data from 25 industrial production companies was used for the present study. In total, 16,285 workers participated in a health check between 2013 and 2018, enriched with data on type of shift schedule, from company records. We excluded participants without data on shift work (*n* = 1223), those with a specific irregular shift schedule in which relatively few participants work (e.g. 2-shift workers, off-shore workers) (*N* = 1025), former shift workers (*N* = 314), or those with missing data on overweight (*N* = 5655), lifestyle (*N* = 560) or covariates (*N* = 94). This resulted in a study population of 4848 non-shift workers and 2569 shift workers. The Medical Ethics Committee of the VU University Medical Center Amsterdam approved the study.

### Shift work

The Human Resources department of the companies provided for each worker information on the type of shift work, i.e. 3-shift work, 4-shift work, 5-shift work, and non-shift work. In general, the 3-shift schedule consisted of a slow-forward rotating schedule of a week morning shift (e.g. from 6:00 to 14:00) followed by a week afternoon (e.g. from 14:00 to 22:00) and night shifts (e.g. from 22:00 to 6:00), with 2 days off during the weekend. The 5-shift schedule consisted of a fast-forward rotating schedule of two morning shifts, two afternoon shifts, and two night shifts, followed by 3 or 4 days off work. The 4-shift schedule also alternated between two or three morning, afternoon and night shifts. The 3-, 4-, and 5-shift schedules were combined into one category, as very few participants had a 3- or 4-shift schedule and all these shift schedules rotated between morning, afternoon, and night shifts. For the majority of the workers the start date of engaging in shift work was provided by the companies, and was used to calculate the number of years of exposure to shift work. For other workers (*N* = 545, 21%), the number of years engaging in employment at the current company was used as a proxy for number of years worked in shift work because change from non-shift to shift work in the same company rarely happened in these production companies.

### Measurements

Body weight and body height were objectively measured by trained assistants/nurses. Overweight was defined as a body mass index (BMI) ≥ 25 kg/m^2^ and obesity as a BMI ≥ 30 kg/m^2^. Data on lifestyle factors were collected using a standardized questionnaire. Participants were asked for how many days of the week during a normal weekday they had performed leisure-time moderate-to-vigorous intensity physical activity (LTPA) for at least 30 min per day. In line with the physical activity guidelines that recommends to meet at least 150 min/week of LTPA (Piercy et al. 2018; Weggemans et al. 2018), LTPA was dichotomized into < 5 days/week and ≥ 5 days/week. Participants were also asked for how many days in the week they had performed on average vigorous intensity physical activity (VPA) for at least 20 min. Based on the physical activity guidelines that recommend VPA for at least two days a week (Piercy et al. 2018; Weggemans et al. 2018), VPA was dichotomized into < 2 days/week and ≥ 2 days/week. Participants were asked how much per day and on how many days of the week they usually had eaten fruits and vegetables. We standardized fruit and vegetable intake as the number of servings per day, and 77 g of vegetables was considered to be one serving, as calculated in a previous study (He et al. 2006). Fruit and vegetables intake was dichotomized as low intake (< 3 portions/day) and high intake (≥ 3 portions/day) (He et al. 2006). Smoking was asked for with a single question and dichotomized into current smoker and non-smoker. Sleep was measured by calculating the average of the scores on the following four statements (Cronbach’s *α* 0.88): last month I (1) had the feeling not to be able to close an eye; (2) slept restless; (3) had difficulty falling asleep; and (4) woke up tired. Answer options ranged from never (score ‘0’) to daily (score ‘4’) on a 5-point Likert scale. This variable was dichotomized at the 75 percentile into good (score < 2.5) and poor (score ≥ 2.5) sleep quality.

Age, gender, education (intermediate secondary education or less vs intermediate vocational or higher secondary education vs higher vocational education or university), working hours/week, and children living at home (yes vs. no/not applicable) were self-reported. Type of work tasks was assessed using the question “*What type of occupation/work do you have?”*, with three response option mainly physically demanding work tasks, mainly mentally demanding work tasks, or a combination of the two.

### Data analysis

The relationship between shift work and being overweight was estimated using logistic regression analysis adjusted for age, gender, education, children living at home, working hours/week, and type of work tasks. The moderating role of lifestyle was assessed using multiplicative and additive interaction (synergism). Multiplicative interaction by lifestyle was assessed using interaction terms between shift work and each lifestyle factor, and by stratifying all analyses by each lifestyle factors (unhealthy vs healthy). Additive interaction was assessed by calculating the Relative Excess Risk due to Interaction (RERI) using the following formula: e^shift work + lifestyle factor + shift work * lifestyle factors ^− e^shift work ^− e^lifestyle factor^ + 1. The 95% confidence intervals were estimated using the delta method (VanderWeele and Knol 2014). If the RERI was not equal to zero, an additive interaction was present. To further explore potential additive interaction, we compared for each lifestyle factor (1) shift workers with a healthy lifestyle; (2) shift workers with an unhealthy lifestyle, and (3) non-shift workers with an unhealthy lifestyle with the reference group (i.e. non-shift workers with a healthy lifestyle).

Multiplicative interaction by age was assessed using an interaction term between shift work and age (continuous), and by stratifying the results at the median age (< 47 years vs ≥ 47 years). Differences in the relationship between shift work and being overweight by years of exposure to shift work were assessed by comparing shift workers working in shift work for (1) < 10 years, (2) 10–19 years, and (3) those ≥ 20 years with non-shift workers. *P* value for trend was estimated by adding the categorical variable (non- shift work, < 10 years, 10–19 years, and ≥ 20 years shift work) to the logistic regression model. To disentangle the interaction between age and years of exposure to shift work on the relationship between shift work and being overweight, we (1) stratified the analyses of years of shift work and being overweight by younger (< 47 years) and older workers (≥ 47 years) and (2) analyzed the multiplicative interaction between shift work and age within all three categories of years exposure to shift work (< 10 years, 10–19 years, and ≥ 20 years shift work). Non-shift workers were included in all three categories.

In sensitivity analyses, we performed all analyses for obesity as outcome instead of overweight to investigate whether the results differed across different levels of overweight. We also performed sensitivity analyses in which we excluded women and those with a 3- or 4-shift system, because there were only few of such individuals. All analyses were performed using Stata/SE, version 14.1 (StataCorp LLC, College Station, Texas), and a two-sided *p* value < 0.05 was considered statistically significant.

## Results

The mean age of the study population was 45.8 years old (SD 10.6), and most participants were men (87%). Almost all shift workers worked in 5-shift schedules. Compared to non-shift workers, shift workers were more often men (81% vs. 98%), low educated (9% vs. 27%), and had more often jobs with mainly physical demands (12% vs. 29%) (Table 1). Shift workers were also more often overweight, obese and more often had unhealthy lifestyle behaviors than non-shift workers.

**Table 1:** Characteristics of the study population

	Non-shift worker *N* = 4848	Shift worker *N* = 2569
Demographics		
Age (years)	45.9 ± 10.4	45.6 ± 10.9
Gender (male)	3933 (81%)	2504 (98%)
Educational level		
Low	431 (9%)	696 (27%)
Intermediate	1678 (35%)	1743 (68%)
High	2739 (57%)	130 (5%)
Living with children	2789 (58%)	1398 (54%)
Work-related		
Type of job		
Physical	603 (12%)	748 (29%)
Mental	3671 (76%)	391 (15%)
Combination	574 (12%)	1430 (56%)
Working hours per week	39.1 ± 6.6	34.4 ± 4.3
Years worked in shifts	NA	19.3 ± 9.8
Type of shift worker		
3-shift worker	NA	28 (1%)
4-shift worker	NA	24 (1%)
5-shift worker	NA	2,517 (98%)
Anthropometry		
BMI	26.0 ± 3.8	27.2 ± 3.9
Overweight/obesity	2,673 (55%)	1,794 (70%)
Obesity	732 (15%)	603 (24%)
Lifestyle		
Sleep quality (poor)	1211 (25%)	937 (36%)
Smoking (yes)	726 (15%)	844 (33%)
Fruit and Vegetables (< 3 portions)	2251 (46%)	1600 (62%)
MVPA (30 min for < 5 days/week)	2716 (56%)	1482 (58%)
VPA (20 min for < 2 days/week)	2278 (47%)	1208 (47%)

Values represent means ± standard deviations, percentages and (numbers)

*BMI* body mass index, *MVPA *moderate-to-vigorous physical activity at work and in leisure-time, *NA *not applicable, *VPA *Vigorous physical activity in leisure-time

### Moderating role of lifestyle

Shift workers had a 1.53 (95% CI 1.33 1.76) higher odds of being overweight than non-shift workers (Table 2). In all strata of the healthy and unhealthy lifestyle factors, shift work was significantly related to being overweight with ORs ranging between 1.32 and 1.74 (Table 2). There was neither multiplicative nor additive interaction in the relationship between shift work and being overweight by level of sleep quality, amount of fruit and vegetables intake, or physical activity level (*p* ≥ 0.05) (Tables 2, 3). The relationship between shift work and being overweight differed significantly by smoking status (*p* for interaction < 0.01). The OR of the relationship between shift work and being overweight was larger among non-smokers (OR 1.67, 95% CI 1.42 1.98) than smokers (OR 1.32, 95% CI 1.00 1.73). There was also negative additive interaction between shift work and smoking (RERI − 0.44, 95% CI − 0.74 − 0.13), which means that the estimated joint effects of being shift worker and non-smoker on overweight was greater than the sum of the estimated OR’s (Table 3). Non-smoking shift workers had a 1.73 (95% CI 1.47 2.03) higher odds of being overweight compared to non-smoking non-shift workers, whereas smoking shift workers had no increased odds of being overweight.

**Table 2:** Multivariable-adjusted odds ratios for differences in the relationship between shift work and being overweight stratified by unhealthy and healthy lifestyle behaviors

	*N*	Odds ratio (95% CI)	*p*-for interaction
Non-stratified relationship	7417	**1.53 (1.33 1.76)**	–
Sleep quality			0.32
Poor	2148	**1.40 (1.08 1.82)**	
Good	5269	**1.57 (1.33 1.86)**	
Smoking			** < 0.01**
Yes	1570	**1.32 (1.00 1.73)**	
No	5847	**1.67 (1.42 1.98)**	
Fruit and vegetables			0.27
< 3 pieces/day	3220	**1.54 (1.28 1.86)**	
≥ 3 pieces/day	4197	**1.50 (1.21 1.85)**	
Physical activity			0.16
< 5 days/week 30 min MVPA	4198	**1.44 (1.18 1.76)**	
≥ 5 days/week 30 min MVPA	3219	**1.53 (1.25 1.87)**	
Fitness norm			0.08
< 2 day/week 20 min VPA	3486	**1.33 (1.09 1.64)**	
≥ 2 days/week 20 min VPA	3931	**1.74 (1.43 2.11)**	

Analyses adjusted for age, gender and education, children living at home, working hours/week, type of work tasks. Boldface indicates statistical significance (*p* < 0.05)

*CI *confidence interval, *MVPA* moderate-to-vigorous physical activity at work and in leisure-time, *VPA* vigorous physical activity in leisure time

**Table 3:** Multiple-adjusted joint relationships of shift work and lifestyle on being overweight

	Non-shift worker	Shift worker	
	Odds ratio (95% CI)	Odds ratio (95% CI)	RERI (95% CI)
Sleep quality			− 0.07 (− 0.31 0.16)
Poor	**1.10 (0.96 1.27)**	**1.55 (1.29 1.87)**	
Good	**Ref**	**1.57 (1.35 1.85)**	
Smoking			− **0.44 (**− **0.74 **− **0.13)**
Yes	0.94 (0.80 1.12)	1.15 (0.95 1.38)	
No	**Ref**	**1.73 (1.47 2.03)**	
Fruit and vegetables			− 0.01 (− 0.24 0.21)
< 3 pieces/day	**1.24 (1.10 1.39)**	**1.78 (1.50 2.10)**	
≥ 3 pieces/day	**Ref**	**1.62(1.34 1.96)**	
Physical activity			0.06 (− 0.21 0.33)
< 5 days/week 30 min MVPA	**1.51 (1.34 1.70)**	**2.09 (1.76 2.49)**	
≥ 5 days/week 30 min MVPA	**Ref**	**1.62 (1.35 1.93)**	
Fitness norm			− 0.11 (− 0.35 0.14)
< 2 day/week 20 min VPA	**1.22 (1.08 1.37)**	**1.69 (1.41 2.02)**	
≥ 2 days/week 20 min VPA	**ref**	**1.68 (1.41 1.99)**	

Boldface indicates statistical significance (*p* < 0.05)

*CI *confidence interval, *MVPA* moderate-to-vigorous physical activity at work and in leisure-time, *VPA* vigorous physical activity in leisure time, *RERI* relative excess risk due to interaction

### Interaction by age and years of shift work

Multiplicative interaction of age was present: the relationship between shift work and being overweight was significantly stronger with older age (*p* for interaction = 0.01) (Table 4). Among younger workers, shift workers had a 1.31 (95% CI 1.07 1.60) higher odds of being overweight, and among older workers this OR was 1.70 (95% CI 1.39 2.08). The relationship between shift work and being overweight was also stronger with more years of exposure to shift work (*p* for trend < 0.001), with an OR of 1.66 (95% CI 1.40 1.97) of being overweight for those working in irregular shifts for ≥ 20 years. Table 5 shows that in younger as well as in older workers, more years of exposure to shift work was related to a higher odds of being overweight (*p* for trend < 0.05). In each category of years of exposure to shift work, a higher age was also related to a stronger relationship between shift work and being overweight (Multiplicative interaction: p for interaction < 0.05).

**Table 4:** Multiple-adjusted odds ratios (95% confidence intervals) for the relationship between shift work and being overweight, stratified by years of exposure to shift work and age

	Odds ratio (95% CI)	*p* for trend	*p* for interaction
Stratified by years of shift work			
Non-shift worker	Ref		
< 10 years	**1.31 (1.07 1.61)**		
10–19 years	**1.56 (1.23 1.96)**		
≥ 20 years	**1.66 (1.40 1.97)**		
Years of shift work		** < 0.001**	
Stratified by age			
Age < 47 years	**1.31 (1.07 1.60)**		
Age ≥ 47 years	**1.70 (1.39 2.08)**		
Shift work*age			**0.01**

Boldface indicates statistical significance (*p* < 0.05)

*CI* confidence interval

**Table 5:** Multivariable-adjusted odds ratios for the relationship between years of exposure to shift work and being overweight, stratified by age

	Age < 47 years		Age ≥ 47 years		
	Odds ratio (95% CI)	*p* for trend	Odds ratio (95% CI)	*p* for trend	*p* for interaction shift work*age
Non-shift workers	Ref		Ref		
< 10 years shift work	**1.30 (1.02 1.66)**		1.42 (0.89 2.27)		**0.04**
10–20 years shift work	1.24 (0.94 1.63)		**2.36 (1.39 4.01)**		**0.001**
≥ 20 years shift work	**1.47 (1.06 2.03)**		**1.69 (1.37 2.09)**		**0.02**
Years of shift work		**0.02**		** < 0.001**	

Boldface indicates statistical significance (*p* < 0.05)

*CI* confidence interval

### Sensitivity analyses

None of the lifestyle factors moderated the relationship between shift work and being obese (Supplemental Table 1). Although the OR of the relationship between shift work and being obese was 1.25 among smokers and 1.47 among non-smokers, this was not a statistically significant difference (*p* for interaction = 0.23) (Supplemental Table 1). The odds of obesity increased with more years of exposure to shift work (*p* for trend < 0.001), but age was not a significant moderator in the relationship between shift work and being obese (*p* for interaction = 0.50) (Supplemental Tables 2–3). Exclusion of women and those working in a 3- or 4-shift system showed results comparable to the main analyses (Supplemental Tables 4–7).

## Discussion

Shift workers had a 1.5 times higher odds of being overweight than non-shift workers. In contrast to our hypothesis, level of physical activity, fruit and vegetables intake, and sleep quality did not moderate the relationship between shift work and being overweight. Smoking was a significant moderator, with the relationship between shift work and overweight being stronger among non-smokers compared to smokers. Although age was not a significant moderator in the relationship between shift work and being obese, we confirmed our hypothesis that age as well as years of exposure to shift work moderated the relationship between shift work and being overweight.

In line with previous research, the present study showed a higher prevalence of overweight among shift workers compared to non-shift workers (Proper et al. 2016; Sun et al. 2018; van Drongelen et al. 2011). We additionally explored the moderating role of lifestyle behaviors in the relationship between shift work and being overweight. Although healthy behaviors have been shown to increase shift work tolerance and to improve circadian rhythms (Atkinson and Davenne 2007; Atkinson et al. 2008; Costa 2003; Froy 2010; Gabriel and Zierath 2019; Mendoza 2007), the present study could not confirm the potential moderating role of level of physical activity, amount of fruits and vegetables intake, and sleep quality. In particular, physical activity and diet were measured using relatively general measures and, therefore, do not capture the full physical activity and dietary patterns. It is consequently still possible that other aspects of physical activity and dietary patterns moderate the relationship between shift work and being overweight. However, in another study, we showed that shift workers were more often physical inactive, ate less fruits and vegetables, and had poorer sleep quality than non-shift workers, and, thereby, these unhealthy lifestyle behaviors mediated the relationship between shift work and being overweight/obese (Hulsegge et al. 2019a, b). Thus, healthy lifestyle behaviors may as moderators not ‘protect’ against the negative effects of shift work on overweight. An unhealthy lifestyle may only play a mediating role by explaining part of the relationship between shift work and overweight.

This study observed that the relationship between shift work and being overweight was stronger in non-smokers than smokers (multiplicative interaction) and that being non-smoker in combination with being shift worker was more strongly related with overweight than the sum of both single relationships (additive interaction). This means that non-smoking shift workers had the highest chance of being overweight, and that smoking seemed to mitigate the relationship between shift work and being overweight. This can partly be explained by findings of previous studies showing that smokers are less often overweight than non-smokers, and that people gain weight after smoking cessation (Aubin et al. 2012; Chiolero et al. 2008; Tian et al. 2015). The moderating role of smoking was less clear for the relationship between shift work and being obese. This relationship with obesity was also stronger in non-smokers than smokers, but this difference was not statistically significant. Future research is needed to further explore the potential moderating role of smoking in the relationship between shift work and being obese, and the reasons why the relationship between shift work and being overweight is particularly strong among non-smokers. To explain the observed moderating role of smoking, it might be of interest to investigate differences between smokers and non-smokers in genetic predisposition and circulating levels of inflammatory markers that affect circadian clock function (Labrecque and Cermakian 2015). It may also be relevant to investigate whether smokers are more likely to smoke than eat at inappropriate times, because mistimed eating has delirious effects on metabolic health (Challet 2019).

Our study provided new findings that further improve the understanding of the impact of age and years of exposure to shift work on the relationship between shift work and being overweight. We showed that age and years of exposure to shift work, independently from each other, moderated the relationship between shift work and being overweight. The moderating role of years of exposure to shift work reflects the dose–response relationship between longer exposure to unhealthy work times and overweight, with our study indicating that shift work may already be related to overweight when it is done for less than 10 years. This is different for obesity, as our results showed that the relationship between shift work and being obese was only present in people who had worked in shifts for more than 10 years. The moderating role of age might be explained by a decreased ability to adjust to changes in circadian rhythms. This makes the circadian system more prone to desynchronization induced by shift work, which in turn may increase the risk of overweight (Hofman and Swaab 2006; Kecklund and Axelsson 2016). In addition, older shift workers have been found to experience more sleep problems than younger shift workers (Hulsegge et al.^ 2019^; Park et al. 2000; Parkes 2002a), which in turn may result in body weight gain (Patel and Hu 2008). In contrast to overweight, the relationship between shift work and being obese was not moderated by age in the present study. This is in line with a study among offshore personal with 12-h shifts rotating between seven days, seven nights and 14 days off. Years of exposure to offshore work was a more important moderator than age in the relationship between shift work and self-reported BMI in that study (Parkes 2002b). Overall, the relationship between shift work and being overweight seems strongest in older workers, but this moderating role of age may differ by degree of overweight and potentially by population or type of shift schedule.

The strength of the present study is the use of a large homogeneous group, which minimizes residual confounding related to differences across companies, such as the organizational culture of companies. For the majority of the participants, objective data on shift work schedule and history of shift work were used. For a small proportion of the shift workers (21%), we used years engaging in employment at the current company as a proxy for number of years worked in irregular shifts. We expect this to have had little impact on the results as very few people change from non-shift work to shift work within the same production company, and additional analyses excluding the workers without objective data on shift work history returned similar results (data not shown). Workers with overweight or obesity may less likely start shift work and may more often leave shift work compared to healthy workers (Nabe-Nielsen et al. 2008). Although we observed significant relationships between shift work and being overweight and obese, this common methodological problem in shift work research may have underestimated these relationships. The main reason for excluding participants was the lack of information on body weight and body height. This had probably little impact on the results, because missing data was due to the companies’ choice not to offer medical examinations to their employees and was neither due to self-selection bias nor to non-response bias of invitees. The little or no impact of the selection is supported by our findings of similar sociodemographic and lifestyle characteristics among those with complete and those without data on overweight (data not shown), and by additional analyses, in which all missing values were set as overweight. These analyses returned virtually the same results as the main analyses (data not shown). Generalization of the results is limited to male blue-collar workers with rotating shift schedules that include night shifts, which is an important group of workers who represent a large part of the total shift work population with high risks of developing chronic diseases (Gan et al. 2015; McMenamin 2007; Parent-Thirion et al.^ 2016^; Proper et al. 2016; Statistics 2018; Sun et al. 2018; van Drongelen et al. 2011).

In conclusion, lifestyle behaviors did not moderate the relationship between shift work and being overweight, except for smoking. The relationship between shift work and being overweight was stronger among non-smokers compared to smokers. More years of exposure to shift work was, independently from age, related to being overweight and obese, with the strongest relationship with overweight among those working in irregular shifts for more than 20 years. Independently from years of exposure to shift work, age moderated the relationship between shift work and being overweight, but not between shift work and being obese. Based on the present study findings, prevention of shift work-related overweight may need to have special attention for non-smoking and older shift workers, as well as for those potentially working in irregular shifts for several decades. The finding that shift workers had a less healthy lifestyle than non-shift workers highlights the need for preventive efforts to promote a healthy lifestyle among shift workers in production companies.

## Electronic supplementary material

Below is the link to the electronic supplementary material.

Supplementary file1 (DOCX 29 kb)

## Data Availability

Due to ethical restrictions related to participant consent and high sensitivity of the company data, all relevant data are under the conditions of HumanTotalCare available upon request to the responsible senior manager Research & Business Development at HumanTotalCare: Heleen Paagman (email: heleen.paagman@arboned.nl).
