# Supplementary material for: The moderating role of lifestyle, age, and years working in shifts in the relationship between shift work and being overweight
Source: Int Arch Occup Environ Health. 2020 Feb 10;93(6):697–705. doi: 10.1007/s00420-020-01519-4 (PMC7320962; doi:10.1007/s00420-020-01519-4)
Supplement: Supplementary file 1 — Supplementary file1 (DOCX 29 kb) [file 420_2020_1519_MOESM1_ESM.docx]

**Supplemental table 1**. Multivariable-adjusted odds ratios for differences in the relationship between shift work and being obese stratified by unhealthy and healthy lifestyle behaviors.

|  | N  Obese/non-obese | Odds ratio  (95%CI) | p for interaction | RERI  (95%CI) |
| --- | --- | --- | --- | --- |
| Non-stratified relationship | 7,417 | **1.37 (1.16 1.61)** |  |  |
|  |  |  |  |  |
| Sleep quality |  |  | 0.74 | 0.04 (-0.14 0.22) |
| Poor | 2,148 | **1.38 (1.03 1.85)** |  |  |
| Good | 5,269 | **1.34 (1.09 1.63)** |  |  |
|  |  |  |  |  |
| Smoking |  |  | 0.23 | -0.38 (-0.79 0.03) |
| Yes | 1,570 | 1.25 (0.87 1.80) |  |  |
| No | 5,847 | **1.47 (1.22 1.76)** |  |  |
|  |  |  |  |  |
| Fruit & vegetables |  |  | 0.44 | 0.01 (-0.15 0.17) |
| <3 pieces/day | 4,197 | **1.31 (1.05 1.62)** |  |  |
| ≥3 pieces/day | 3,220 | **1.43 (1.11 1.85)** |  |  |
|  |  |  |  |  |
| Physical activity |  |  | 0.58 | 0.13 (-0.02 0.27) |
| <5 days/week 30 min MVPA | 4,198 | **1.40 (1.09 1.81)** |  |  |
| ≥5 days/week 30 min MVPA | 3,219 | **1.27 (1.02 1.58)** |  |  |
|  |  |  |  |  |
| Fitness norm |  |  | 0.39 | 0.12 (-0.04 0.28) |
| <2 day/week 20 min VPA | 3,486 | 1.17 (0.94 1.46) |  |  |
| ≥2 days/week 20 min VPA | 3,931 | **1.73 (1.35 2.11)** |  |  |

CI: confidence interval; MVPA: moderate-to-vigorous physical activity at work and in leisure-time; VPA: vigorous physical activity in leisure time.

RERI: relative excess risk due to interaction.

Boldface indicates statistical significance (p< 0.05).

**Supplemental Table 2.** Multiple-adjusted odds ratios (95% confidence intervals) for the relationship between shift work and being obese, stratified by years of exposure to shift work and age.

|  | Odds ratio (95%CI) and p-values for interaction |
| --- | --- |
| **Stratified by years of shift work** | |
| Non-shift worker | ref |
| <10 years | 1.05 (0.80 1.38) |
| 10-19 years | **1.40 (1.07 1.83)** |
| ≥20 years | **1.49 (1.24 1.78)** |
| P-value for linear trend | **<0.001** |
|  |  |
|  |  |
| **Stratified by age** |  |
| Age <47 years | 1.24 (0.95 1.62) |
| Age ≥47 years | **1.35 (1.09 1.67)** |
| P-value interaction shift work*age | 0.50 |

CI; confidence interval.

Boldface indicates statistical significance (p< 0.05).

**Supplemental Table 3**. Multivariable adjusted odds ratios for the relationship between years of exposure to shift work and being obese, stratified by age.

|  | Age <47 years | Age ≥47 years |  |
| --- | --- | --- | --- |
|  | Odds ratio (95%CI) | Odds ratio (95%CI) | p-value interaction shift work*age |
| Non-shift workers | ref | ref |  |
| <10 years shift work | 1.14 (0.81 1.60) | 0.99 (0.59 1.66) | 0.37 |
| 10-20 years shift work | **1.41 (1.00 1.98)** | **1.01 (0.68 1.81)** | 0.87 |
| ≥20 years shift work | 1.29 (0.90 1.83) | **1.42 (1.14 1.76)** | **0.03** |
|  |  |  |  |
| P-value for trend for years exposure to shift work | 0.08 | **0.001** |  |

Boldface indicates statistical significance (p< 0.05).

**Supplemental Table 4**. Multivariable-adjusted odds ratios for differences in the relationship between shift work and being overweight stratified by unhealthy and healthy lifestyle behaviors among male non-shift and 5-shift workers.

|  | N | Odds ratio (95%CI) | | p-for interaction |
| --- | --- | --- | --- | --- |
| Non-stratified relationship | **6,390** | **1.63 (1.40 1.98)** | |  |
|  |  |  |  |  |
| Sleep quality |  |  |  | 0.42 |
| Poor | **1,770** | **1.63 (1.22 2.18)** | |  |
| Good | **4,620** | **1.61 (1.35 1.92)** | |  |
|  |  |  |  |  |
| Smoking |  |  |  | **0.006** |
| Yes | 1,381 | 1.35 (0.999 1.81) | |  |
| No | **5,009** | **1.80 (1.51 2.16)** | |  |
|  |  |  |  |  |
| Fruit & vegetables |  |  |  | 0.20 |
| <3 pieces/day | **3,676** | **1.55 (1.26 1.90)** | |  |
| ≥3 pieces/day | **2,714** | **1.71 (1.36 2.15)** | |  |
|  |  |  |  |  |
| Physical activity |  |  |  | 0.21 |
| <5 days/week 30 min MVPA | **3,567** | **1.53 (1.24 1.90)** | |  |
| ≥5 days/week 30 min MVPA | **2,823** | **1.62 (1.30 2.00)** | |  |
|  |  |  |  |  |
| Fitness norm |  |  |  | 0.07 |
| <2 day/week 20 min VPA | **2,981** | **1.40 (1.12 1.75)** | |  |
| ≥2 days/week 20 min VPA | **3,409** | **1.88 (1.53 2.31)** | |  |

CI: confidence interval; MVPA: moderate-to-vigorous physical activity at work and in leisure-time; VPA: vigorous physical activity in leisure time.

Analyses adjusted for age, gender and education, children living at home, working hours/week, type of work tasks. Boldface indicates statistical significance (p< 0.05).

**Supplemental Table 5.** Multiple-adjusted joint relationships of shift work and lifestyle on being overweight among male non-shift and 5-shift workers.

|  | Non-shift worker | | Shift worker | |  |  |
| --- | --- | --- | --- | --- | --- | --- |
|  | Odds ratio (95%CI) | | Odds ratio (95%CI) | | RERI (95%CI) | |
| Sleep quality |  |  |  |  | -0.05 (-0.28 0.19) | |
| Poor | 1.11 (0.95-1.29) | | **1.67 (1.37 2.04)** | |  |  |
| Good | ref |  | **1.67 (1.41 1.98)** | |  |  |
|  |  |  |  |  |  |  |
| Smoking |  |  |  |  | **-0.46 (-0.79 -0.13)** | |
| Yes | 0.97 (0.80-1.18) | | **1.24 (1.02 1.51)** | |  |  |
| No | ref |  | **1.85 (1.56 2.19)** | |  |  |
|  |  |  |  |  |  |  |
| Fruit & vegetables |  |  |  |  | -0.01 (-0.24 0.23) | |
| <3 pieces/day | **1.26 (1.40 1.43)** | | **1.90 (1.58 2.28)** | |  |  |
| ≥3 pieces/day | ref |  | **1.75 (1.43 2.14)** | |  |  |
|  |  |  |  |  |  |  |
| Physical activity |  |  |  |  | 0.12 (-0.15 0.39) | |
| <5 days/week 30 min MVPA | **1.50 (1.31 1.71)** | | **2.21 (1.84 2.65)** | |  |  |
| ≥5 days/week 30 min MVPA | ref |  | **1.70 (1.84 2.65)** | |  |  |
|  |  |  |  |  |  |  |
| Fitness norm |  |  |  |  | -0.09 (-0.35 0.17) | |
| <2 day/week 20 min VPA | **1.24 (1.09 1.42)** | | **1.82 (1.50 2.20)** | |  |  |
| ≥2 days/week 20 min VPA | ref |  | **1.80 (1.50-2.16)** | |  |  |

CI: confidence interval; MVPA: moderate-to-vigorous physical activity at work and in leisure-time; VPA: vigorous physical activity in leisure time; RERI: relative excess risk due to interaction.

Boldface indicates statistical significance (p< 0.05).

**Supplemental Table 6.** Multiple-adjusted odds ratios (95% confidence intervals) for the relationship between shift work and being overweight, stratified by years of exposure to shift work and age among male non-shift and 5-shift workers.

|  | Odds ratio (95%CI) | | p for trend | p for interaction |  |
| --- | --- | --- | --- | --- | --- |
| **Stratified by years of shift work** | |  |  |  |  |
| Non-shift worker | ref |  |  |  |  |
| <10 years | **1.39 (1.12 1.72)** | |  |  |  |
| 10-19 years | **1.67 (1.30 2.14)** | |  |  |  |
| ≥20 years | **1.75 (1.47 2.09)** | |  |  |  |
| Years of shift work |  |  | **<0.001** |  |  |
|  |  |  |  |  |  |
| **Stratified by age** |  |  |  |  |  |
| Age <47 years | **1.44 (1.15 1.79)** | |  |  |  |
| Age ≥47 years | **1.74 (1.41 2.15)** | |  |  |  |
| Shift work*age |  |  |  | **0.03** |  |

CI: confidence interval.

Boldface indicates statistical significance (p< 0.05).

**Supplemental Table 7**. Multivariable-adjusted odds ratios for the relationship between years of exposure to shift work and being overweight, stratified by age among male non-shift and 5-shift workers.

|  | Age <47 years | | |  | Age ≥47 years | | |  | |
| --- | --- | --- | --- | --- | --- | --- | --- | --- | --- |
|  | Odds ratio (95%CI) | | p for trend |  | Odds ratio (95%CI) | | p for trend | p for interaction shift work*age |  |
| Non-shift workers | Ref |  |  |  | Ref |  |  |  | |
| <10 years shift work | **1.44 (1.11 1.87)** | |  |  | 1.32 (0.81 2.15) | |  | 0.13 | |
| 10-20 years shift work | 1.32 (0.98 1.78) | |  |  | **3.05 (1.66 5.62)** | |  | **<0.001** | |
| ≥20 years shift work | **1.63 (1.15 2.30)** | |  |  | **1.72 (1.38 2.14)** | |  | **0.008** | |
| Years of shift work |  |  | **0.005** |  |  |  | **<0.001** |  | |

CI: confidence interval.

Boldface indicates statistical significance (p< 0.05).
